# The Impact of COVID-19 Pandemic on Patients Receiving Orthodontic Treatment

**DOI:** 10.1055/s-0041-1742130

**Published:** 2022-03-08

**Authors:** Zaid Shhabat, Mo'ath Ghozlan, Nadeem Kana'an, Ashraf Tashtoush, Ayman Alelaimat, Rami Saadeh

**Affiliations:** 1 Royal Medical Services, Irbid, Jordan; 2 Department of Public Health and Community Medicine, Faculty of Medicine, Jordan University of Science and Technology, Irbid, Jordan

**Keywords:** Jordan, COVID-19, Treatment, Orthodontic, Appointment, Lockdown

## Abstract

**Objective** The coronavirus disease 2019 (COVID-19) pandemic impacted the provision of dental treatment in a timely manner including orthodontic treatment. The objective of this study was to assess challenges encountered by patients undergoing orthodontic treatment during the pandemic and their attitude toward managing these challenges.

**Material and Methods** A cross-sectional sample of orthodontic patients at Prince Rashid Hospital in the north of Jordan was recruited to participate in a self-administered questionnaire that included questions related to orthodontic problems encountered during the lockdown, ways to overcome these problems, and concerns about their treatment.

**Results** A total of 120 patients participated, 40 males and 80 females. Most participants ( *n* = 86) had a fixed orthodontic appliance, who reported various orthodontic problems (82%), but the most common problems reported were exposed wire endings and loosening of brackets. Likewise, the most commonly reported problem among those with removable appliances ( *n* = 34) was a broken or loose appliance (60%). However, patients of both types of appliances ignored the problem and waited until the re-opening of dental offices. Further, most patients missed at least one appointment for different reasons and three-quarters of them (76.6%) did not communicate with their orthodontists during the lockdown. Patients expressed their disagreement with the closure of dental offices during the pandemic because of their concern of a prolonged waiting to receive another appointment.

**Conclusion** The COVID-19 pandemic had a significant impact on orthodontic care and the maintenance of appointments during the lockdown periods. Patients varied in ways they managed their orthodontic problems, including their commitment to communicate with the dentists or maintain their appointments. Hence, patients should be trained on how to manage the appliance problems when they need and encourage them to accept distant communication during the emergencies.

## Introduction

The novel coronavirus disease 2019 (COVID-19) broke out in Wuhan, China, at the end of 2019 and, then, spread globally, causing major health, humanitarian, and financial crises. 1 Each affected country has adopted different policies and strategies to combat the pandemic, and many clinics closed partly or completely to control the infection in the absence of vaccines. 2 Dental offices were no exception, especially since the main route of the transmission of the virus is through the respiratory tract, direct or close contact with infected persons, and the asymptomatic carriage of the virus, which could increase the possibility of transmission. 3

Despite the recommendations released on methods of protection and proper precautions to follow, 4 many governments preferred to lockdown their countries with a complete closure for unnecessary health care. 5 Oral and dental care during the lockdown considered only urgent care under strict precautions, and all other routine procedures were postponed, causing a massive delay in receiving care and compensating for patients' appointments. 6

Orthodontic treatment requires a regular visit over a long period that could reach 2 years and the COVID-19 pandemic affected patients' treatment schedule and disrupted the success of a quality treatment because of prolonged and missed appointments. 1 7 According to Xiong et al 2020, 90.39% of orthodontic patients had their appointments delayed for over a month. 8 Beckwith et al reported that orthodontic treatment was prolonged for 1.09 months with each missed appointment. 9 During this pandemic and subsequent clinic closures, most of the orthodontic patients were unable to get the care they needed, causing anxiety and psychological distress. Hence, the purpose of this study was to evaluate the challenges faced by orthodontic patients at prince Rashid Hospital in the north of Jordan during the COVID-19 pandemic, ways they managed these challenges, and concerns about their orthodontic treatment.

## Material and Methods

### Study Design and Settings

This was a cross-sectional study conducted on orthodontic patients who attended the orthodontic clinic at Prince Rashid Hospital in the north of Jordan after the lockdown was lifted.

### Sampling

A self-administered questionnaire was handed to a total of 130 patients who visited the orthodontic clinic to receive treatment. Patients were approached on random days during the first month of re-opening of clinics after the lockdown (May 2020). During their waiting time, the main investigator of the study talked to patients in the waiting room about the purpose of the study and method of participating, explaining the optionality of participation, confidentiality, and privacy of information. Those who consented and successfully completed the self-administered questionnaire were 120, forming a response rate of 92.3%. No interruption during filling the questionnaire or assistance in explaining questions was provided to participants to avoid any bias toward certain answers and ensure that the investigators' opinions are not imposed on their answers.

### Instrument

The questions of the questionnaire were developed after reviewing related studies and adopted questions that meet the study objectives. 4 10 11 The questionnaire consisted of four parts. The first part included personal information (age, gender, and type of appliance), the second part was related to patients' attitude during the closure, the third part discussed problems encountered and their management, and the last part was related to patients' concerns about their orthodontic treatment.

The questions were first developed in English and checked by the investigator and another researcher for their comprehensibility and adaptability with study objectives. A translator was asked to translate the questionnaire into Arabic and another one to translate it back into English to check the proficiency and correctness of the translation. The main investigator and the other researcher checked the Arabic version before it was provided to participants.

### Ethical Considerations

This study gained approval from the internal review board of the Royal Medical Services in Jordan.

## Results

Although 130 patients were outreached to participate in the study, 120 were included in the analysis and 10 respondents were excluded due to inconsistencies of answers they provided to repeated questions and some incomplete answers.

The mean age of the participants was 18.5 years, including 40 (33.3%) males and 80 (66.7%) females. Patients who had fixed appliances (FA) were 86, divided into 26 males and 60 females, while those who had removable appliances (RA) were 34, divided into 14 males and 20 females.

Table 1 illustrated the attitude of patients toward their orthodontic treatment during the lockdown. Three out of each four patients (76.6%) did not communicate with their orthodontists during the lockdown, while only 6.6% communicated three or four times. In addition, over 60% of them disagreed with the closure of dental clinics and had missed their appointments for over a 2-month period. This is reflected on 68% who responded that they stopped visiting the orthodontic clinic because the clinic was closed and not because they feared the COVID-19 spread (9.2%).

**Table 1:** Patients attitude during the closure of clinics

	Item	%
How many times did you communicate with your orthodontist during the pandemic?	One to twice Three times Four times Did not communicate	20 (16.7%) 4 (3.3%) 4 (3.3%) 92 (76.7%)
To what extent do you agree or disagree with closing dental clinics to minimize the spread of COVID-19?	Agree Partially agree Neutral Do not agree Strongly disagree	16 (13.3%) 9 (7.5%) 22 (18.3%) 30 (25%) 43 (35.8%)
What was the reason you stopped visiting your orthodontic?	Clinic was closed I was afraid of the COVID-19 spread Both reasons Other reasons	82 (68.3%) 11 (9.2%) 22 (18.3%) 5 (4.2%)
How long was the period during which you missed the appointments during the pandemic?	Did not miss any 1–2 mo 2–3 mo More than 4 mo	20 (16.7%) 24 (20.0%) 30 (25.0%) 46 (38.3%)

Abbreviation: COVID-19, coronavirus disease 2019.

When patients were asked about their main concern regarding the closure of clinics during the pandemic, 52.5% of them were worried about a prolonged orthodontic treatment period, 41.7% were concerned about unexpected occurrence of problems including a problem with their appliances, and only 5.8% were afraid that the orthodontist will get busy and will have no time no treat them after clinics re-open.

Of 86 FA patients, 82.6% faced problems with their devices. The main problem encountered was an exposed wire end (34.9%), followed by bracket and band loosening (16.6%), and other problems ( Table 2 ). Patients managed these problems differently: 49.3% ignored the problem, 14.1% requested an emergency appointment, 14.1% used orthodontic wax, 21.1% searched for a solution on the internet, and 1.4% sent a picture to their orthodontist, as demonstrated in Table 3 . In comparison, of 34 RA patients, over half of them (55.9%) had problems during the pandemic, and most of these problems were a broken appliance (44.1%) or a loose appliance (32%). Similarly, most RA patients managed their orthodontic problems by ignoring them (52%) or by looking for a solution on the internet (34.3%).

**Table 2:** Problems encountered during the closure of clinics

Fixed appliance	Removable appliance
No problem (17.4%) Exposed wire end (34.9%) Bracket and band off (16.6%) Gum swelling (11.6%) Broken metal piece (9.0%) Loss of spring (4.7%) Pain (2.3%) Appearance of space (2.3%) Run out of elastic band (1.2%)	No problem (14.7%) Broken part of the appliance (44.1%) Loose appliance (32.3%) Gum swelling (5.9%) Appearance of spaces (3.0%)

**Table 3:** Ways patients managed their orthodontic problems during the closure of clinics

Fixed appliance	Removable appliance
Ignored the problem (49.3%) Requested an emergency appointment (14.1%) Used orthodontic wax (14.1%) Searched on the internet for solution (21.1%) Sent a picture to their orthodontist (1.4%)	Ignored the problem (52.0%) Searched on the internet for solution (34.3%) Requested emergency appointment (6.9%) Sent a picture to their orthodontist (3.4%) Called the orthodontist (3.4%)

Table 4 shows patients' concerns regarding their orthodontic treatment. Most patients were worried about the suspension of their regular visits to the orthodontist (79.2%) and 60% thought that it should be considered as an emergency. Only 13.3% did not think it is a real emergency, while the rest thought that certain problems should only be considered emergencies.

**Table 4:** Patients concerns about their orthodontic treatment

Are you worried about stopping regular orthodontic visits during the pandemic?	Worried ( *n* = 95, 79.2%) Not worried ( *n* = 25, 20.8%)
Do you think that orthodontic treatment should be considered an emergency?	Yes, because I do not want anything to go wrong with my treatment ( *n* = 72, 60.0%). Only certain problems can be considered emergencies, such as cuts, lacerations, swellings, etc. ( *n* = 32, 26.7%) No, because it is not life threatening ( *n* = 16, 13.3%)

## Discussion

The lockdown of dental clinics during the COVID-19 pandemic has represented a major problem for orthodontic patients who usually need to be seen regularly by their orthodontists. Hence, this study aimed to evaluate the problems faced by orthodontic patients, their concerns, and ways they dealt with their problems, which shall help to avoid such problems in the future. In addition, exploring factors related to delayed or incomplete dental services pour in the advantage of the overall health of patients. Low levels of oral health care do not only lead to poor oral health behaviors but rather poor general health behaviors, 12 which is considered a serious consequence to delayed oral health services.

Problems reported by patients with an FA were different than those reported by patients with RA. FA patients reported more problems, and the most common problem encountered was irritation from the wire ending due to the friction between the end of the wire and the inner cheek or the tongue. The second most commonly reported problem among FA patients was a loose band or bracket (band/bracket off). These results were close to those found by Shenoi et al 2020, 10 showing that the main two problems faced by the patients were loosening of brackets, followed by pain from exposed wire end. This is similar to orthodontic problems reported by Gyawali et al 2019 13 and Jones et al 2016 14 indicating the most common reason for orthodontic emergency to be loosening of brackets. On the contrary, the most commonly reported problem by RA patients was a broken part of the device and the least common problem was appearing of space.

Most patients of both types of appliances responded to their problems by ignoring them, as reported by 49.3% of FA and 52% of RA patients. This high percentage indicates either poor compliance and motivation or a low level of awareness about the importance of maintaining their appliances. Searching for a solution on the internet was adopted by one-fifth of FA patients (21%) and one-third of RA patients (34.4%), possibly because the internet has become an alternative way to find information and learn from the experience of other people, who have experienced the same situation. Only few patients of both types of appliances tried to outreach to the orthodontist by calling, sending a picture, or requesting an urgent appointment.

A major problem was postponing of appointments. More than 63% had their appointments postponed for more than 2 months, which is much higher than what was published by Shenoi et al 2020, 10 showing that 48% of the patients had missed their appointments for the same period. This could explain why most patients in the current study were worried about the duration of which the clinics would stay closed (79.2%). A close percentage was reported by Shenoi et al 2020, 10 demonstrating that three-fourths (73.5%) of patients were worried about the same matter. However, this was higher than what Xiong et al. 2020 had reported. 8 Patients in the current study were not worried about getting infected during the visits and considered their orthodontic treatment as an emergency, which, therefore, made them to request a close rescheduling for an appointment. Although these concerns about the closure of clinics seemed significant, most of them (76.7%) did not communicate with their orthodontist during the pandemic and ignored their problem. A possible explanation is that patients felt inconvenient to an alternative solution and believed in physical presence in the orthodontic clinic. These findings were contrary to other studies which reported that most patients found a way to contact their doctors, indicating a different perception and probably a higher level of awareness than patients in the current study. 15 16 However, it is reported that lengthy treatment courses could result in less compliance by patients, 17 which was clear in the current study, showing only 14.1% of patients asked for help from their doctors due to the extended treatment course. As a comparison, Shenoi et al 2020 reported a higher percentage (49.0%). 10 These delayed periods of dental appointments were not a concern of patients alone but was a serious concern for dentists as well who were afraid to lose their patients after the quarantine phase and significant economic damage could result to their business if similar quarantine periods happen again. 18

Considering these results, more attention should be paid to orthodontic treatment plans because of the long treatment course. Orthodontists should better use the simplest treatment plan with the shortest course and least complicated appliances as possible, which should help in any lockdown or pandemic in the future. In addition, patients should be educated on how to manage their dental problems if they are not able to seek a dental office for help.

## Conclusion

The COVID-19 pandemic had a significant impact on orthodontic care and the maintenance of appointments during the lockdown periods. Patients using fixed and RA reported a variety of problems, with more frequent problems occurring among patients with FA. Educating orthodontic patients on managing their appliance problems during any lockdown periods by feasible ways is imperative. Besides, alternative ways of communication between orthodontic patients and their dentists should be applied especially during disasters or health pandemics. One way is using telehealth consultations, which seems promising and supportive to the health care system 19 and could help orthodontists to outreach their patients, 20 since many patients do not have an interest in distant communication and become less compliant with lengthy procedures.
